# Pyrimidine synthesis inhibition enhances cutaneous defenses against antibiotic resistant bacteria through activation of NOD2 signaling

**DOI:** 10.1038/s41598-018-27012-0

**Published:** 2018-06-07

**Authors:** Samreen Jatana, Craig R. Homer, Maria Madajka, András K. Ponti, Amrita Kabi, Francis Papay, Christine McDonald

**Affiliations:** 1 Department of Inflammation and Immunity, Lerner Research Institute, Cleveland Clinic, Cleveland, Ohio USA; 2 Department of Plastic Surgery, Dermatology and Plastic Surgery Institute, Cleveland Clinic, Cleveland, Ohio USA; 3 Department of Molecular Medicine, Cleveland Clinic Lerner College of Medicine, Case Western Reserve University, Cleveland, Ohio USA

## Abstract

Multidrug-resistant bacterial strains are a rapidly emerging healthcare threat; therefore it is critical to develop new therapies to combat these organisms. Prior antibacterial strategies directly target pathogen growth or viability. Host-directed strategies to increase antimicrobial defenses may be an effective alternative to antibiotics and reduce development of resistant strains. In this study, we demonstrated the efficacy of a pyrimidine synthesis inhibitor, *N*-phosphonacetyl-l-aspartate (PALA), to enhance clearance of methicillin-resistant *Staphylococcus aureus* (MRSA)*, Pseudomonas aeruginosa*, and *Acinetobacter baumannii* strains by primary human dermal fibroblasts *in vitro*. PALA did not have a direct bactericidal effect, but enhanced cellular secretion of the antimicrobial peptides human β-defensin 2 (HBD2) and HBD3 from fibroblasts. When tested in porcine and human skin explant models, a topical PALA formulation was efficacious to enhance MRSA, *P. aeruginosa*, and *A. baumannii* clearance. Topical PALA treatment of human skin explants also resulted in increased HBD2 and cathelicidin (LL-37) production. The antimicrobial actions of PALA required expression of nucleotide-binding, oligomerization domain 2 (NOD2), receptor-interacting serine/threonine-protein kinase 2 (RIP2), and carbamoyl phosphatase synthase II/aspartate transcarbamylase/dihydroorotase (CAD). Our results indicate that PALA may be a new option to combat multidrug-resistant bacterial infections of the skin through enhancement of an integral pathway of the cutaneous innate immune defense system.

## Introduction

A major healthcare problem is the rapidly expanding prevalence of multidrug-resistant (MDR) bacterial strains^1^. Over 2 million people in the United States acquire serious infections with MDR strains each year, and approximately 23,000 individuals die from these infections^2^. The loss of effective antimicrobial treatments has significant impact on routine medical care; common infections now often require more toxic, expensive, and less efficacious antibiotics. This significantly affects not only vulnerable populations, such as immune comprised individuals, the elderly, infants, and those with diabetes or other chronic health conditions, but also healthy individuals undergoing surgical procedures that require effective protection against secondary infection. With the alarming increase in antibiotic resistance, there is an unmet clinical need to design novel and innovative methods to treat MDR bacterial infections.

Skin is frequently colonized with polymicrobial populations that belong to the ESKAPE family, including *Enterococcus faecium, Staphylococcus aureus, Klebsiella pneumoniae, Acinetobacter baumannii, Pseudomonas aeruginosa and Enterobacter spp*^3,4^. These organisms are opportunistic pathogens and are the leading cause of nosocomial infections^2,5^. ESKAPE pathogens are characterized by a high rate of antimicrobial resistance that is acquired through multiple mechanisms that include: drug inactivation, modification of drug binding sites, increased drug efflux, and enhanced biofilm formation^5^. These pathogens often cause a local infection through a breach in the skin. Even in otherwise healthy children and adults, many of these cases progress to fatal, invasive infections or severe, life-threatening conditions, such as necrotizing fasciitis, pneumonia, septicemia, and endocarditis.

Skin is the first line of defense in the human body against invading pathogens^6^. It presents both a physical and chemical barrier to invasion through the presence of a hydrophobic stratum corneum and the constitutive expression of several classes of antimicrobial peptides (AMPs), such as cathelicidin and β-defensins^7^. When this barrier is breached due to wounds, cuts, or abrasions, cells present in the skin epidermis and dermis play important roles in amplifying protective defenses and generating specific immune responses^8–10^. Pattern recognition receptors, like Toll-like receptors (TLRs) and NOD-like receptors (NLRs), are key components of the innate immune response that sense the infection and initiate signaling that result in the secretion of chemokines, cytokines, and AMPs^11^. These secreted products act to provide immediate antimicrobial activity, activate resident cells, and recruit additional immune cells to clear the infection^12,13^. There is increasing interest in augmenting or enhancing these innate immune responses to prevent or treat bacterial infections, as this strategy would not further contribute to the rise of MDR strains and could potentially be combined with other antimicrobial therapies^14,15^.

In a previous study, we identified a small molecule compound that enhances the antimicrobial activity of the pattern recognition receptor, nucleotide-binding, oligomerization domain 2 (NOD2)^16^. NOD2 is an intracellular sensor of both intracellular and extracellular bacterial pathogens, including *Salmonella, S. aureus, A. baumannii*, and *P. aeruginosa*^17–19^. It is expressed in both immune cells and barrier cells of the skin^20^, where it plays critical roles in wound healing and cutaneous defense against pathogens^19,21^. Mice deficient in NOD2 expression (*Nod2*^−/−^) have delayed wound healing due to defective keratinocyte migration and reduced neutrophil recruitment^21^. Additionally, *Nod2*^−/−^ mice infected subcutaneously with *S. aureus* have impaired bacterial clearance and an exacerbated ulcerative response^19^. NOD2 functions in keratinocytes to regulate the expression of AMPs, such as human β-defensin 2 (HBD2) and HBD3, through the activation of an intracellular signaling cascade that requires receptor-interacting serine/threonine-protein kinase 2 (RIP2)^15,22,23^. One challenge to modulating the activity of this pathway is that the NOD2 protein does not have a specific, pharmacologically targetable enzyme activity. However, we identified that NOD2 activity is negatively regulated by the enzyme carbamoyl phosphatase synthase II/aspartate transcarbamylase/dihydroorotase (CAD)^16,24^. CAD is an enzyme required for *de novo* pyrimidine synthesis and can be inhibited by a specific small molecule compound *N*-phosphonacetyl-l-aspartate (PALA)^25^. Our studies in intestinal epithelial cells demonstrated that PALA treatment specifically relieves CAD-mediated repression of NOD2 activity, resulting in enhanced clearance of intracellular bacteria^16^. The current study tests whether this method of NOD2 activation to stimulate innate immune responses could be an effective approach to target antibiotic resistant bacterial infections in skin wounds.

The results of this study show that PALA enhances bacterial clearance in a NOD2 signaling pathway-dependent manner using *in vitro* models. These infection models include studies of primary human dermal fibroblasts, as well as *ex vivo* pig and human skin explants. Results demonstrate that PALA is not directly bactericidal or cytotoxic. Instead, PALA relieves the CAD-mediated inhibition of NOD2 signaling to result in enhanced secretion of antimicrobial peptides that are effective in killing both Gram-positive and Gram-negative bacteria of the ESKAPE pathogen family. Our results indicate that PALA may be a new option to combat multidrug-resistant bacterial infections of the skin through enhancement of an integral pathway of the cutaneous innate immune defense system.

## Results

### PALA is not directly bactericidal, but enhances bactericidal activity of normal human dermal fibroblasts (NHDF)

**Figure 1:**
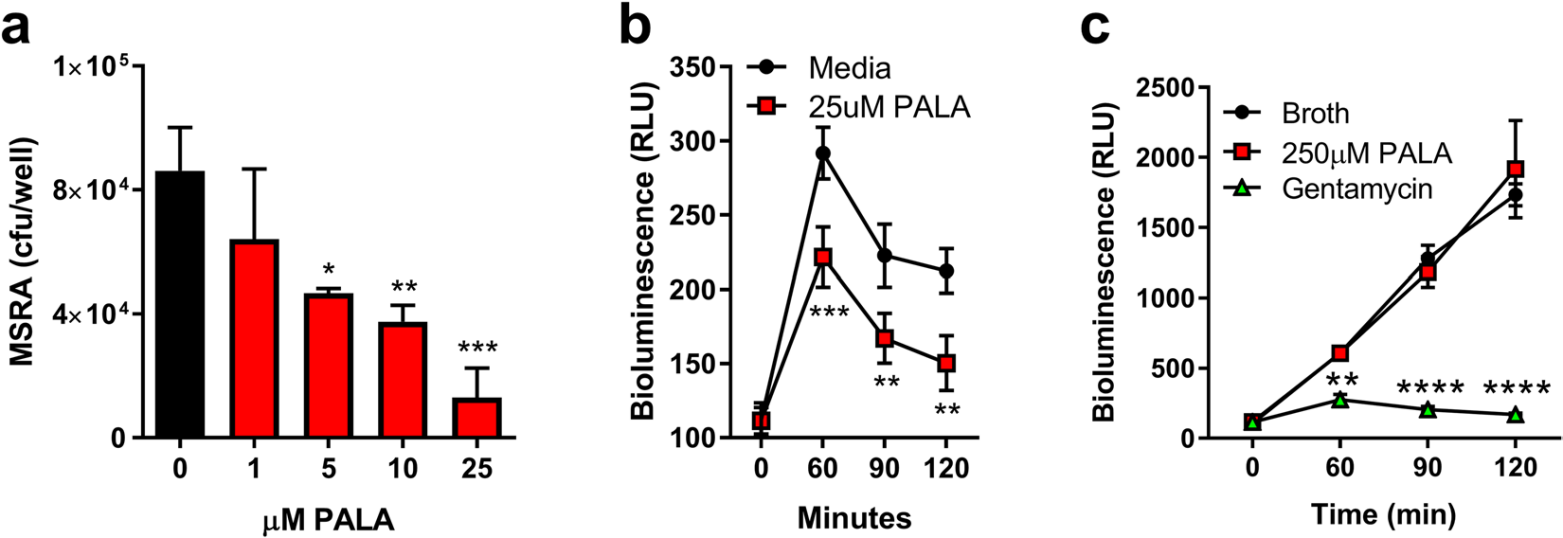
PALA is not directly bactericidal but enhances the bactericidal activity of NHDF. (**a**) PALA treatment reduces MRSA viability in NHDF cultures in a dose-dependent manner. NHDF were treated with 0–25 µM PALA for 16 hours, then infected with bioluminescent MRSA (MOI = 5) in triplicate. After 2 h of infection, cfu were determined. Mean ± SD, n = 5 independent experiments; significance determined by 2-way ANOVA and Bonferroni multiple comparison test; *p < 0.05, **p < 0.01, ***p < 0.001. (**b**) PALA treatment improves the kinetics of MRSA clearance in NHDF cultures. NHDF were treated with or without 25 µM PALA for 16 hours, then infected with bioluminescent MRSA (MOI = 5) in triplicate and bacterial viability monitored over time by bioluminescence levels in triplicate cultures. Mean ± SD, n = 4 independent experiments; significance determined by 2-way ANOVA and Bonferroni multiple comparison test; **p < 0.01, ***p < 0.001. (**c**) PALA is not directly bactericidal to MRSA. Early-log phase cultures of bioluminescent MRSA were unsupplemented (Broth) or supplemented with either 250 µM PALA, or gentamycin (50 µg/mL) and bacterial viability monitored over time by bioluminescence levels of triplicate cultures. Mean ± SD, n = 3 independent experiments; significance determined by 2-way ANOVA and Tukey’s multiple comparisons test; **p < 0.01, ****p < 0.0001.

Several pattern recognition receptors including NOD2 are expressed in skin and contribute to host defenses against *S. aureus*^19^. Therefore, we examined whether PALA treatment is effective in generating antimicrobial responses against methicillin-resistant *S. aureus* (MRSA) in primary fibroblasts derived from human skin (Fig. 1). NHDF were treated with up to 25 µM PALA for 16 hours, and then infected with a bioluminescent MRSA strain^26^, followed by quantification of colony forming units (cfu). MRSA viability was dose-dependently decreased by PALA treatment of NHDF cultures, with significant differences observed at concentrations of 5, 10, and 25 µM (Fig. 1a). The kinetics of bacterial clearance in NHDF cultures was measured by quantification of MRSA bioluminescence over time in the presence and absence of PALA. NHDF were pre-treated with 25 µM PALA for 60 minutes, then infected with bioluminescent MRSA, and bioluminescence monitored over 120 minutes. PALA-mediated enhancement of MRSA clearance was observed within 60 minutes and was maintained at 90 and 120 minutes (Fig. 1b). These results demonstrate that PALA decreases MRSA viability rapidly and in a dose-dependent manner.

PALA inhibits the aspartate transcarbamylase (ATC) activity of CAD to prevent *de novo* pyrimidine synthesis^25^. Bacteria also express ATC; therefore, we investigated whether the decreased MRSA cfu observed after PALA treatment is due to direct bactericidal or bacteriostatic effects (Fig. 1c). Early log-phase cultures of bioluminescent MRSA were supplemented with either PALA (250 µM) or gentamycin (50 µg/ml) and growth monitored over 120 minutes. MRSA grown in unsupplemented broth and PALA supplemented cultures showed similar increases in bioluminescent signal, indicating robust bacterial growth (Fig. 1c). In contrast, cultures supplemented with the antibiotic gentamycin did not grow. These results indicate that PALA, even at doses 10-times higher than an effective concentration to induce a cellular antimicrobial response, does not have direct bactericidal or bacteriostatic activity.

### PALA is not cytotoxic and does not inhibit wound healing responses in NHDF

**Figure 2:**
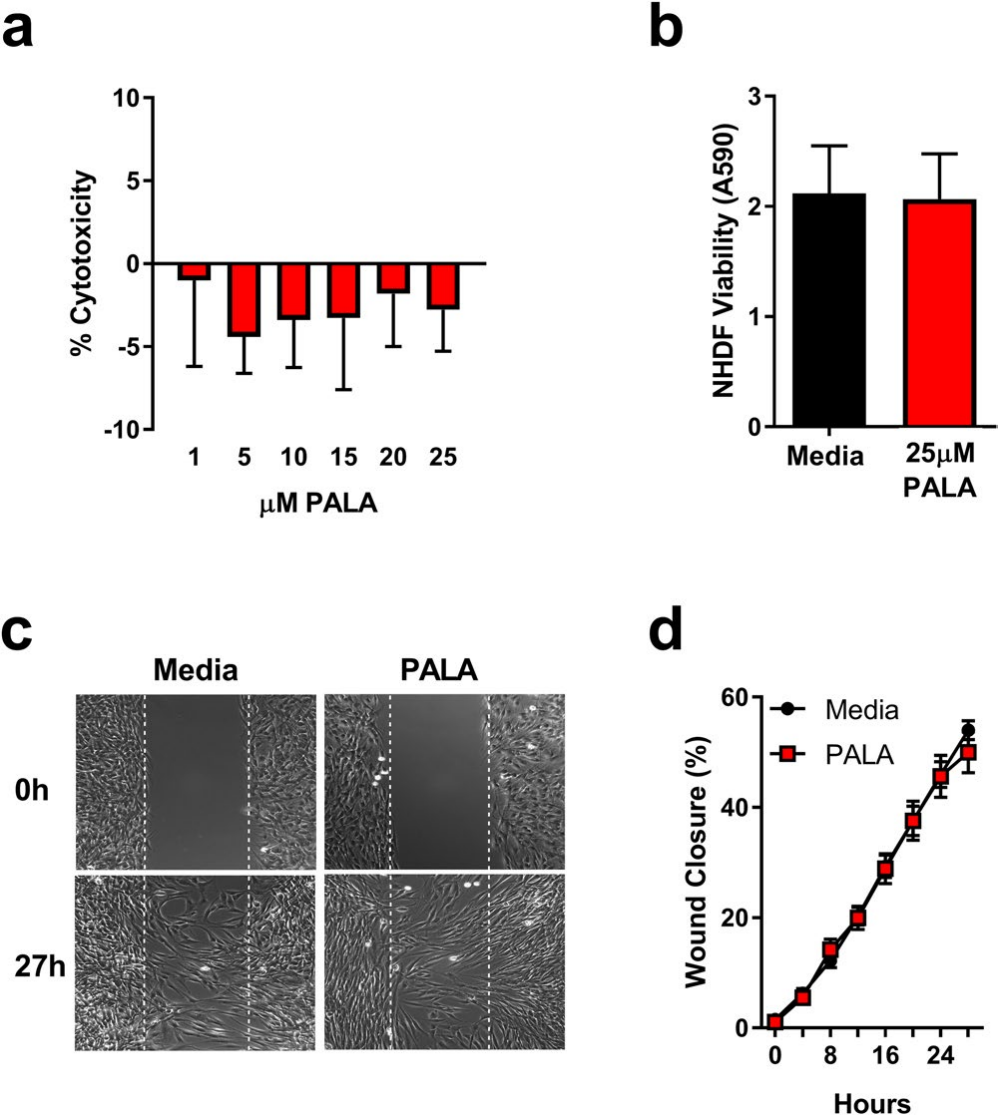
PALA is not cytotoxic and does not inhibit wound healing responses in NHDF. (**a**) Cellular viability is unaltered after treatment with 1–25 µM PALA in triplicate for 24 hours as assessed in by LDH release assay in triplicate (n = 3 independent experiments). (**b**) Cellular proliferation is not affected by PALA (25 µM; 24 hours) as determined by XTT assay in triplicate (n = 3 independent experiments). (**c**) PALA (25 µM) does not affect NHDF migration in scratch wound assays. Representative images from 0 h and 27 hours post-wounding shown. Wound area marked by dashed lines. Individual experiments performed in triplicate wells and images representative of 3 independent experiments. Full time course movies shown in Supplemental Movies S1 and S2. **(d)** Quantitation of live cell image analysis of the effects of PALA on NHDF migration in scratch wound assays. Repeated measurements of wound area from triplicate wells of three independent experiments. Mean ± SD; significance determined by 2-way ANOVA and Bonferroni multiple comparison test.

One concern about the therapeutic use of a pyrimidine synthesis inhibitor, such as PALA, is the potential for cytotoxic effects on host cells. To determine the cytotoxicity of PALA, NHDF cells were exposed to a range of PALA concentrations shown to induce an antibacterial effect (0–25 µM) for 24 hours and cell viability determined using the lactate dehydrogenase (LDH) release assay. No change in cell viability was observed at the highest concentration of PALA tested (25 µM), a dose that is most effective in clearing bacteria from the cells (Fig. 2a). The impact of PALA on cellular proliferation was also measured by an XTT cell proliferation assay. No difference in cellular growth was observed between untreated and PALA (25 µM) treated NHDF after 24 hours (Fig. 2b). Additionally, the effects of PALA on NHDF cell migration were assessed by live cell imaging of *in vitro* scratch wound assays to determine if PALA may inhibit wound healing responses. No significant differences in cell migration were observed between untreated and PALA-treated NHDF over the 27 hour time course (Fig. 2c,d; Supplemental Movies S1 and S2). These findings indicate that PALA rapidly induces an antibacterial response to MDR bacterial infection at a non-cytotoxic concentration in primary human skin-derived cells.

### PALA treatment results in clearance of ESKAPE pathogens and production of broad-spectrum AMPs by NHDF

**Figure 3:**
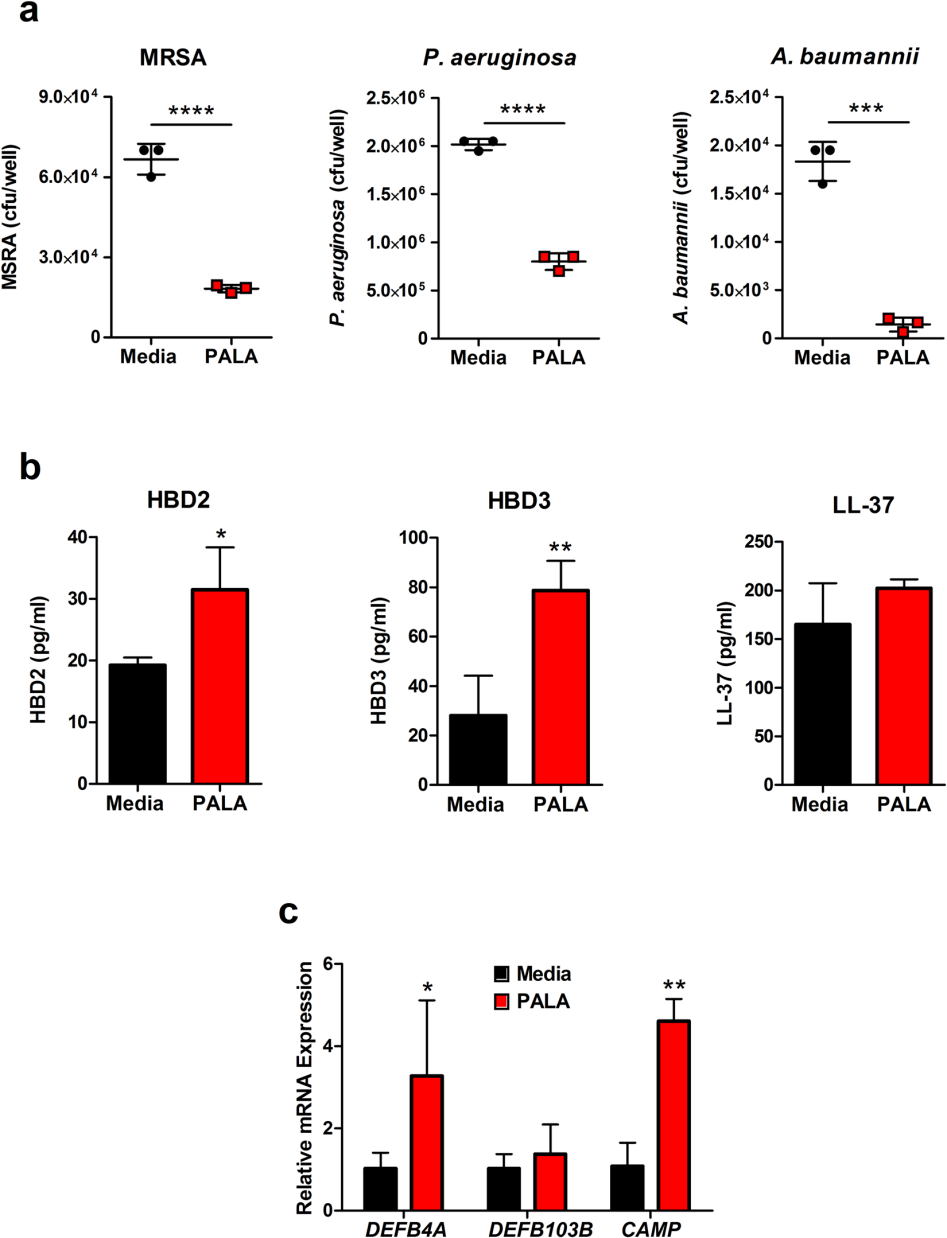
PALA treatment results in the secretion of broad-spectrum AMPs from NHDF that kill ESKAPE pathogens. (**a**) Conditioned supernatants from PALA treated NHDF reduce viability of three pathogens of the ESKAPE family. Triplicate mid-log phase cultures of MRSA, *P. aeruginosa*, and *A. baumannii* were supplemented with conditioned supernatants collected from NHDF treated with or without PALA (25 µM, 16 hours) and cfu determined after 2–4 hours. Each symbol shown represents mean values of an independent experiment performed in triplicate. Results representative of 6 independent experiments. Mean ± SD; significance determined by unpaired, 2-tailed, Students t-test; ***p < 0.001, ****p < 0.0001. (**b**) PALA treatment induces the secretion of AMPs from NHDF. AMP levels in conditioned media collected from triplicate cultures of NHDF treated with or without PALA (25 µM, 16 hours) were quantitated by ELISA. Results representative of 6 independent experiments performed. Mean ± SD; significance determined by unpaired, 2-tailed, Students t-test; *p < 0.05, **p < 0.01. (**c**) Transcription of the genes encoding HBD2 (*DEFB4A*), and cathelicidin (*CAMP*) is enhanced by PALA treatment, while transcripts of the gene encoding HBD3 (*DEFB103B*) are not increased. NHDF were treated with or without 25 µM PALA for 16 hours in triplicate and RNA extracted. Transcript levels relative to unstimulated cells were determined by qRT-PCR from three independent experiments. Mean ± SD; significance determined by 2-way ANOVA and Bonferroni multiple comparisons test; *p < 0.05, **p < 0.01.

In order to characterize the spectrum of antibacterial activity induced by PALA in NHDF, we tested the bactericidal activity of conditioned supernatants on both Gram-positive and Gram-negative bacterial strains of the ESKAPE pathogen family. Mid-log cultures of MRSA, *P. aeruginosa*, and *A. baumannii* were supplemented with conditioned supernatants from NHDF treated with or without PALA and bacterial viability measured by selective plating. Supernatants from PALA treated cells were effective in reducing recovered cfu of all three strains (Fig. 3a), indicating that PALA stimulates the secretion of broad spectrum antimicrobial factors from NHDF.

The major families of AMPs expressed in the skin and induced by bacterial infection are cathelicidins (LL-37) and β-defensins (HBD2 and HBD3)^7^. Therefore, we quantified the levels of these candidate AMPs released into the media from untreated or PALA treated NHDF by ELISA. The protein levels of HBD2 and HBD3, were found to be significantly elevated in supernatants from PALA treated cells, while secreted LL-37 levels were unaffected (Fig. 3b). Further analysis of PALA treated NHDF was performed to determine whether PALA treatment induces transcriptional upregulation of AMPs or primarily stimulates AMP secretion. Transcript levels of *DEFB4A* (HBD2), *DEFB103B* (HBD3), and *CAMP* (cathelicidin) in PALA-stimulated NHDF were quantitated relative to levels in unstimulated cells by qRT-PCR. PALA stimulated the transcription of the genes encoding HBD2 and cathelicidin, but not HBD3 (Fig. 3c). These results demonstrate that in NHDF, PALA primes the production of cathelicidin, increases the release of HBD3, and upregulates both the production and release of HBD2, resulting in an enhanced ability of NHDF to kill both Gram-positive and Gram-negative ESKAPE pathogens.

### Topical application of PALA enhances the clearance of MRSA*, P. aeruginosa and A. baumannii* from skin explants

**Figure 4:**
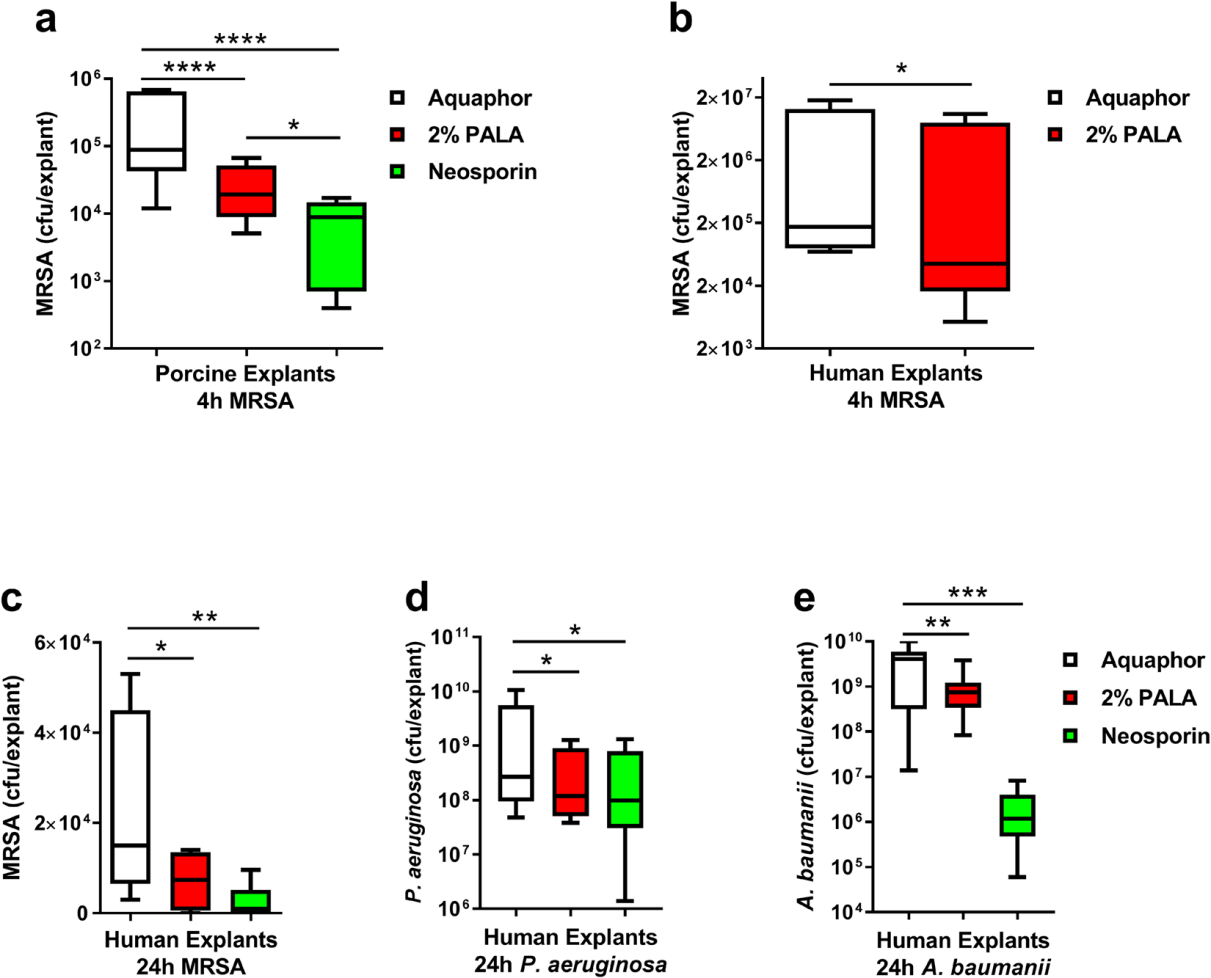
Topical application of PALA enhances the clearance of *S. aureus, P. aeruginosa, and A. baumannii* from skin explants. (**a**) Topical PALA increases MRSA clearance in *ex vivo* pig skin explants. Pig skin biopsies were infected with MRSA for 1 h, followed by topical application of vehicle (Aquaphor), 2% (w/w) PALA, or triple antibiotic ointment (Neosporin) for 4 hours in triplicate. Cfu were determined from biopsy homogenates (n = 6 donors). Box and whiskers plot of min to max values shown; significance determined by 2-way ANOVA and Tukey’s multiple comparisons test; *p < 0.05, ****p < 0.0001. (**b**) Topical PALA increases MRSA clearance in *ex vivo* human skin explants. Human skin biopsies were infected MRSA for 1 h, followed by topical application of vehicle (Aquaphor), or 2% (w/w) PALA for 4 hours in triplicate. Cfu were determined from biopsy homogenates (n = 5 donors). (**c**) Topical PALA increases MRSA, *P. aeruginosa*, and *A. baumannii* clearance in *ex vivo* human skin explants. Human skin biopsies were infected with MRSA, *P. aeruginosa*, or *A. baumannii* for 1 h, followed by topical application of vehicle (Aquaphor), 2%(w/w) PALA, or Neosporin for 24 hours in triplicate. Cfu were determined from biopsy homogenates (n = 5–6 donors). Box and whiskers plot of min to max values shown; significance determined by Wilcoxon matched-pairs signed rank test; *p < 0.05, **p < 0.01, ns = p > 0.05.

In order to translate our findings into a more clinically-relevant context, a topical formulation of PALA was prepared and tested for induction of antibacterial activity in primary skin explants (Fig. 4). Biopsies from fresh skin samples obtained from Yorkshire pigs were maintained *ex vivo* in an air-liquid interface culture and tested within 24 hours of collection. Explants were wounded with a 25 g needle and infected with MRSA for 1 h. Infected biopsies were then treated topically with either Aquaphor (vehicle control), 2% (w/w) PALA, or the triple antibiotic ointment Neosporin for 4 hours. Bacterial load was determined by selective plating of biopsy homogenates. Significantly fewer cfu were recovered from biopsies treated with 2% PALA or Neosporin, as compared to the Aquaphor treated group (Fig. 4a). A similar effect was observed when topical PALA was tested on *ex vivo* human skin explants, where treatment with 2% PALA was significantly more effective in killing bacteria as compared to Aquaphor treated biopsies after 4 hours (Fig. 4b). Excitingly, in addition to MRSA, 2% PALA treatment (24 hours) improved clearance of *P. aeruginosa* and *A. baumannii* by human skin explants (Fig. 4c,d and e). The results indicate that topical application of PALA effectively stimulates an antibacterial response against three different members of the ESKAPE pathogen family in skin samples that results in enhanced bacterial clearance.

### Topical application of PALA enhances AMP production in human skin explants

**Figure 5:**
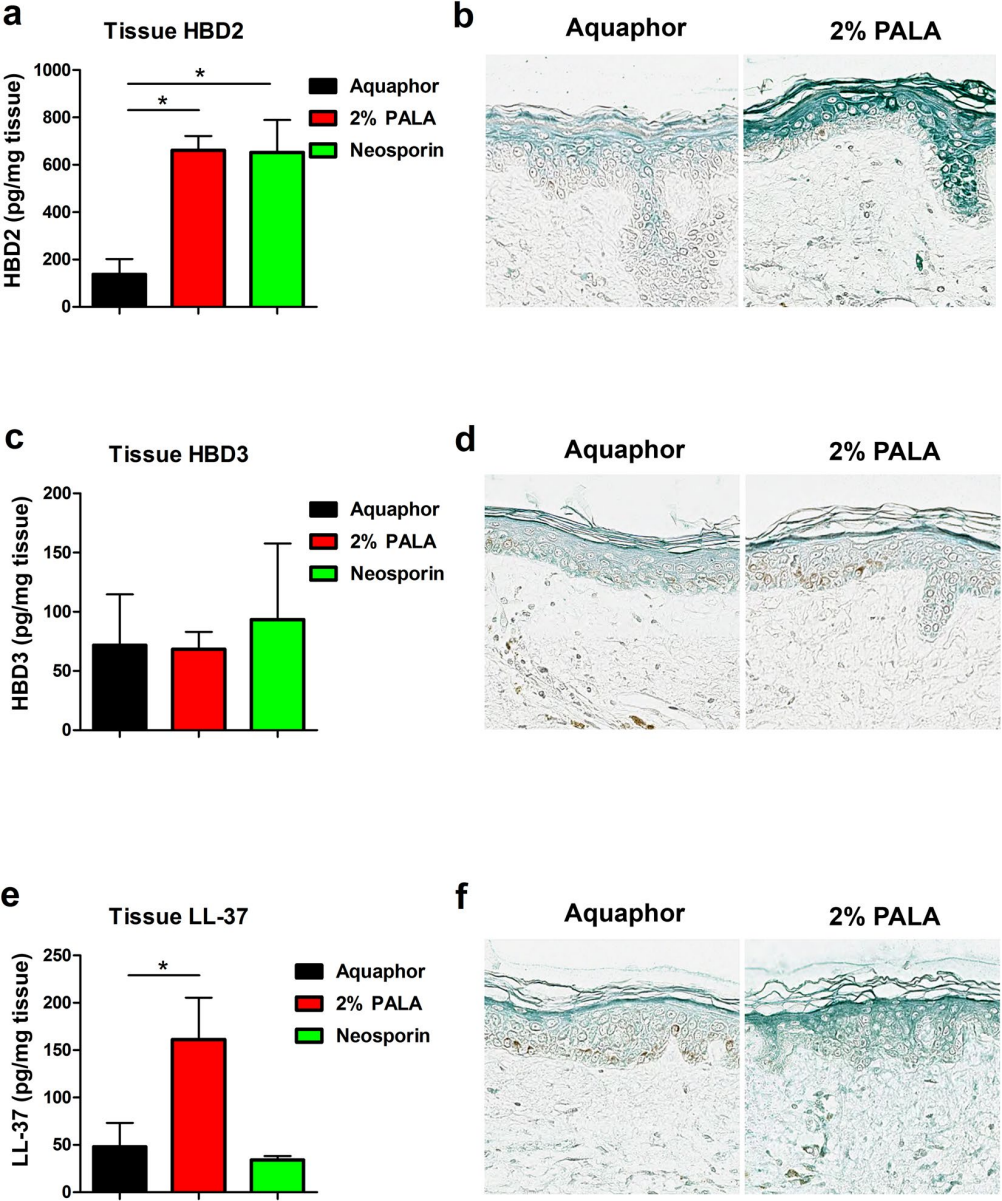
Topical application of PALA enhances AMP production in human skin explants. (**a**) HBD2 protein levels are increased in tissue homogenates of infected human skin explants by PALA or Neosporin treatment. Human skin biopsies were infected with MRSA for 1 h and then treated with the indicated ointments for 24 hours in triplicate. HBD2 levels were measured in tissue homogenates by ELISA (n = 3–4 donors). Mean ± SD; significance determined by 1-way ANOVA and Bonferroni multiple comparison test; *p < 0.05. (**b**) Immunohistochemistry staining of HBD2 in MRSA infected human skin explants described in (**a**). Representative image of 3 donors. (**c**) HBD3 protein levels in tissue homogenates of infected human skin explants are unaffected by PALA or Neosporin treatment. HBD3 levels were measured in tissue homogenates described in (**a**) by ELISA (n = 4 donors). Mean ± SD; significance determined by 1-way ANOVA and Bonferroni multiple comparison test; p > 0.05. (**d**) Immunohistochemistry staining of HBD3 in MRSA infected human skin explants described in (**a**). Representative image of 4 donors. **(e)** LL-37 protein levels are increased in tissue homogenates of infected human skin explants by PALA treatment. LL-37 levels were measured in tissue homogenates described in (**a**) by ELISA (n = 4–5 donors). Mean ± SD; significance determined by 1-way ANOVA and Bonferroni multiple comparison test; *p < 0.05. (**f**) Immunohistochemistry staining of LL-37 in MRSA infected human skin explants described in (**a**). Representative image of 6 donors.

We quantified the levels of candidate AMPs (HBD2, HBD3, and LL-37) in human skin explants treated with Aquaphor, 2% PALA, or Neosporin by ELISA of tissue homogenates. The protein levels of HBD2 and LL-37 were significantly elevated in explants treated with 2% PALA compared to the control (Aquaphor), with no significant differences observed in the HBD3 levels across various treatment groups (Fig. 5a,c and e). HBD2 levels were also significantly elevated by Neosporin treatment as compared to Aquaphor treatment (Fig. 5a). Immunohistochemistry was performed on MRSA infected skin explants to visually detect the level and location of AMP expression in response to topical PALA treatment (Fig. 5b,d,f). Similar to the ELISA results, HBD2 and LL-37 staining was more intense in explants treated with 2% PALA in comparison to Aquaphor, while HBD3 staining was equivalent between treatment groups. HBD2 expression primarily co-localized to the differentiating keratinocyte layers and not the stratum basale (Fig. 5b). HBD3 staining localized throughout the epidermis, and did not change in response to 2% PALA treatment (Fig. 5d). Cathelicidin (LL-37) staining in Aquaphor treated explants was most pronounced in the stratum corneum and stratum lucidum (Fig. 5f). After PALA treatment, LL-37 was detected more widely in the epidermis to include the lucidum, granular, and spinosum layers (Fig. 5f). These results indicate that topical PALA treatment of skin explants enhances the production of HBD2 and LL-37 with broad-spectrum activity, which is effective in killing three different pathogens.

### PALA treatment specifically increases NOD2-dependent *S. aureus* clearance

**Figure 6:**
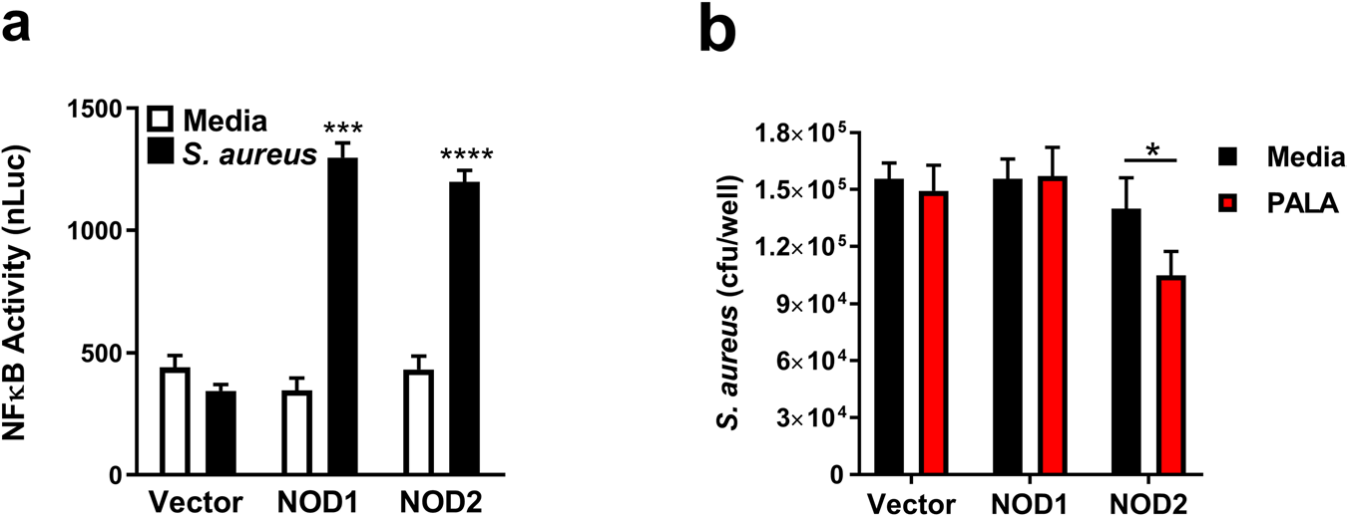
PALA treatment specifically enhances NOD2-dependent secretion of antimicrobial factors that target *S. aureus*. (**a**) *S. aureus* is sensed by intracellular NLRs and stimulates NLR-dependent NFκB activity in reporter gene assays. HEK293T cells were transfected with empty vector, NOD1, or NOD2 expression constructs with NFκB-driven luciferase reporter and β-galactosidase transfection control plasmids. Cells were infected with *S. aureus* Reynolds capsular serotype CP5 (MOI = 5) for 6 h. Triplicate luciferase values were normalized to transfection efficiency measured by β-galactosidase levels (nLuc). Mean ± SD, n = 3 independent experiments; significance determined by 2-way ANOVA and Bonferroni multiple comparison test; ***p < 0.001, ****p < 0.0001. (**b**) PALA specifically enhances NOD2-dependent secretion of antimicrobial factors that target *S. aureus*. HEK293T cells were transfected with empty vector, NOD1, or NOD2 expression constructs and treated with or without 25 µM PALA for 24 hours in triplicate. Mid-log phase cultures of MRSA were supplemented with conditioned supernatants collected from transfected HEK293T cells for 1 h and cfu determined from three independent experiments. Mean ± SD; significance determined by 2-way ANOVA and Bonferroni multiple comparison test; *p < 0.05.

NOD1 and NOD2 are intracellular NLR proteins reported to sense and respond to extracellular pathogens, such as *S. aureus*^19,27,28^. We confirmed that NOD1 and NOD2 can sense *S. aureus* using a combination of luciferase reporter assays and bacterial clearance assays in HEK293T cells (Fig. 6). HEK293T cells do not express NLRs or TLRs endogenously and allow for the study of individual pattern recognition receptor responses when expressed exogenously. HEK293T cells were transfected with empty expression vector, NOD1, or NOD2 expression plasmids, as well as an NFκB-dependent luciferase reporter construct and a constitutive β-galactosidase expression transfection efficiency control. The cells were subsequently infected with *S. aureus*, followed by assessment of NLR-dependent NFκB signaling and antibacterial clearance. Vector transfected cells did not increase NFκB activity in response to *S. aureus* infection, indicating they lacked expression of pattern recognition receptors required to sense this bacterium (Fig. 6a). Exogenous expression of NOD1 or NOD2 resulted in a robust *S. aureus*-induced NFκB activation. These data confirm that both NOD1 and NOD2 are sensors of extracellular *S. aureus* and activate downstream signaling pathways in response to *S. aureus* infection.

Previously, we demonstrated that PALA enhanced the clearance of the intracellular pathogen *Salmonella* in a NOD2-dependent manner^16^. We tested whether PALA also requires NOD2 expression to stimulate the release of antimicrobial factors that target extracellular pathogens, such as *S. aureus*. HEK293T cells were transfected with NOD1 or NOD2 expression plasmids. The transfected cells were treated with or without PALA for 24 hours, and the bactericidal effect of conditioned supernatants was tested on *S. aureus* cultures. NOD2 expression was required for PALA treatment to increase secreted antibacterial activity, as only supernatants from NOD2 expressing cells demonstrated enhanced *S. aureus* killing in response to PALA treatment (Fig. 6b). These experiments confirm that both NOD1 and NOD2 can sense extracellular pathogens, like *S. aureus*, and demonstrate that PALA treatment increases secretion of antibacterial factors that increase bacterial clearance of these organisms in a NOD2-dependent manner.

### The antibacterial effects of PALA require expression of CAD and NOD2 signaling molecules

**Figure 7:**
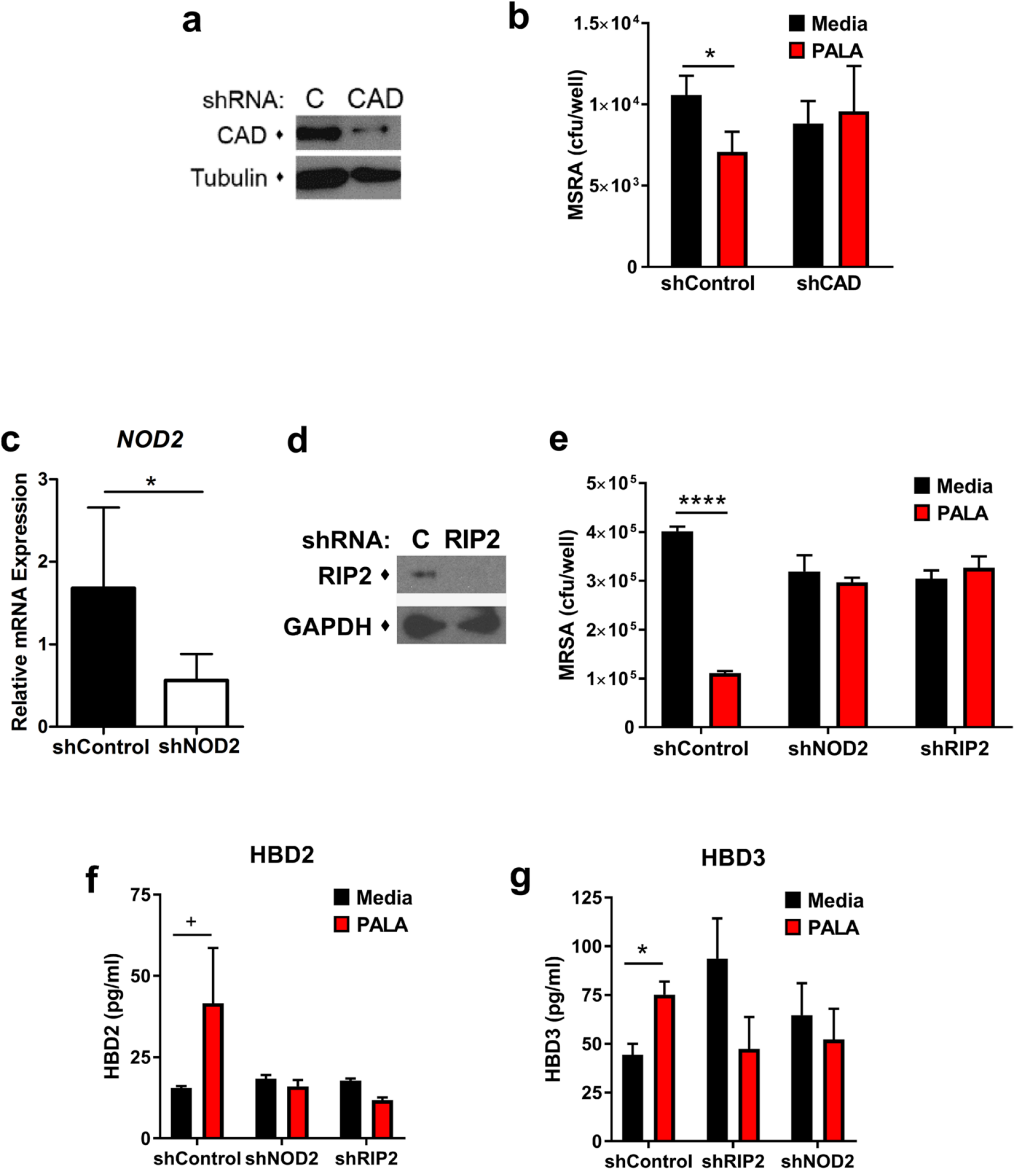
The antibacterial effects of PALA require expression of CAD and NOD2 signaling molecules. (**a**) Immunoblot validation of shRNA-mediated knockdown of CAD in NHDF. NHDF were transfected with non-targeting (C) or CAD-targeting (CAD) shRNA constructs and after 48 h cell lysates were analyzed by immunoblot for CAD. Tubulin levels were also assessed as a loading control. Blots representative of 7 independent experiments. (**b**) CAD expression is required for PALA-stimulated release of antimicrobial factors from NHDF. NHDF were transfected with non-targeting (shControl) or CAD-targeting (shCAD) shRNA constructs. After 48 h, cells were treated with 25 µM PALA (16 hours) and conditioned media collected. Bactericidal activity of conditioned media was determined through addition to mid-log phase MRSA cultures for 2 h in triplicate. (n = 7 independent experiments) Mean ± SD; significance determined by 2-way ANOVA and Bonferroni multiple comparison test; *p < 0.05. **(c)** Validation of shRNA-mediated knockdown of NOD2 expression by qRT-PCR. NOD2 transcripts normalized to 18 S ribosomal transcripts and relative expression of shNOD2 expressing NHDF was determined in comparison to shControl expressing NHDF. Mean ± SD; significance determined by unpaired Student’s t-test; *p < 0.05. (**d**) Immunoblot validation of shRNA-mediated knockdown of RIP2 in NHDF. NHDF were transfected with non-targeting (C) or RIP2-targeting (RIP2) shRNA constructs and after 48 h cell lysates were analyzed by immunoblot for RIP2. GAPDH levels were also assessed as a loading control. Blots representative of 8 independent experiments. (**e**) NOD2 and RIP2 expression are required for the antibacterial effects of PALA treatment in NHDF. NHDF were transfected with the indicated shRNAs and after 24 hours treated with or without 25 µM PALA (24 hours) and conditioned media collected. Bactericidal activity of conditioned media was determined through addition to mid-log phase MRSA cultures for 1 h in triplicate. (n = 4–7 independent experiments) Mean ± SD; significance determined by 2-way ANOVA and Bonferroni multiple comparison test; ****p < 0.0001. (**f**,**g**) RIP2 and NOD2 expression is required for PALA-stimulated release of antimicrobial factors from NHDF. NHDF were transfected with the indicated shRNAs. After 24 hours, cells were treated with 25 µM PALA (24 hours) and conditioned media collected. Protein levels of secreted HBD2 and HBD3 in conditioned media was determined by ELISA in triplicate. (n = 3–5 independent experiments) Mean ± SD; significance determined by 2-way ANOVA with Bonferroni multiple comparisons test; ^+^p = 0.06, *p < 0.05.

Pharmacologic inhibitors often have off-target effects; therefore, we determined the requirement for CAD expression for the antimicrobial effects of PALA stimulation. NHDF were transfected with non-targeting (shControl) and CAD-targeting (shCAD) shRNA constructs and CAD protein levels were determined by immunoblot to confirm RNAi-mediated knockdown of CAD expression (Fig. 7a). Transfected cells were treated with or without PALA for 24 hours and conditioned supernatants added to mid-log phase MRSA cultures to determine bactericidal activity by selective plating. PALA-stimulated antimicrobial activity observed in shControl conditioned supernatants was lost in supernatants from shCAD cells (Fig. 7b). These results indicate that PALA requires CAD expression to induce an antimicrobial response in NHDF.

Next, we investigated whether components of the NOD2 signaling cascade were required for PALA enhanced clearance of MRSA by NHDF. NOD2 signaling requires RIP2, a dual specificity kinase, to activate a signal transduction cascade in response to bacterial infection^15^. NHDF were transfected with shRNA targeting NOD2 (shNOD2), RIP2 (shRIP2), or a non-targeting control (shControl) plasmid and knockdown was confirmed by qRT-PCR or immunoblot (Fig. 7c,d). Transfected cells were subsequently treated with or without PALA for 24 hours and bactericidal activity of the conditioned supernatants assessed on mid-log phase MRSA cultures. PALA treatment of shControl transfected NHDF increased the bactericidal activity of conditioned supernatants (Fig. 7e). In contrast, knockdown of NOD2 or RIP2 resulted in similar levels of bacterial killing between supernatants from untreated and PALA treated cells. Knockdown of NOD2 or RIP2 also impaired the secretion of HBD2 and HBD3 from these cells as determined by ELISA of conditioned supernatants used for bacterial clearance assays (Fig. 7f,g). These results indicate NOD2 and RIP2 expression are required for the antibacterial effects of PALA treatment in NHDF and suggests that NOD2 signaling plays an important role in this response.

## Discussion

Multiple factors have contributed to the rise of MDR pathogens, including the misuse and overuse of antibiotics, supplementation of agricultural feed with antibiotics, the rapid cross-transfer of antibiotic resistance genes between bacteria, as well as a decreased level of antibiotic development in pharmaceutical company pipelines^29,30^. As a result, it is predicted that there will be 300 million premature deaths worldwide due to antimicrobial resistance by 2050 if there is not a dramatic change to our approach to MDR pathogens^31^. In this scenario, deaths from infectious disease are estimated to be 10 million/year, exceeding cancer-related mortality and making it one of the leading causes of death^31^. To avoid this, it will require a multi-faceted approach to infection control that integrates not only additional antibiotics, but also improved infection prevention measures, increased awareness and understanding of MDR, greater education regarding optimal application of antibiotic therapy, and accelerated development of novel antimicrobial agents. This study identifies a small molecule agent, PALA, as new potential addition to the arsenal to combat MDR bacterial infections.

An ever increasing number of studies demonstrate the benefits of stimulating pattern recognition receptors to protect against microbial infection through induction of a broad spectrum antimicrobial response^29,32,33^. One example of this is the administration of flagellin to stimulate TLR5-mediated immune responses, which provided protection against *P. aeruginosa* in a lung infection model^34^. However, one danger of this approach is the potential to exacerbate inflammation and tissue damage through overstimulation of an inflammatory immune response. This was observed in initial studies with TLR4 agonists that included a toxic lipid A component of endotoxin; use of more selective monophosphoryl lipid A (MPLA) or aminoalkyl gluosaminide phosphates (AGPs) are now clinically in use as vaccine adjuvants^33^. Our results indicate that PALA acts to increase basal NOD2 signaling activity by inactivating CAD, a negative regulator of NOD2 (Fig. 7)^16^. Our data also demonstrate that derepression of NOD2 by PALA resulted in increased expression and release of AMPs (Figs 3b,c and 7f,g), while not inducing the release of the pro-inflammatory cytokines IL-1β, IL-6, or TNFα from NHDF (data not shown). While the molecular mechanisms underlying this differential enhancement of NOD2-dependent responses requires further study, this feature of PALA to boost antimicrobial factor production, rather than directly stimulate inflammatory immune responses may increase the specificity and safety of this approach to infection control.

NOD2 is an integral component of the innate immune system and serves as sensor for a wide range of intracellular and extracellular bacteria^11,15^. Studies in *Nod2*^−/−^ mice have shown the importance of NOD2 in maintaining antimicrobial homeostasis in the skin, as well as promoting wound healing responses. *Nod2*^−/−^ mice are more susceptible to subcutaneous infection with *S. aureus* and develop an exacerbated ulcerative response^27^. Additionally, mice deficient in NOD2 expression are impaired in acute wound healing due to a combination of altered skin microbiota^35^, delayed immune cell recruitment to wound sites^21^, and impaired epidermal proliferation and migration^21^. With the key role of NOD2 in mitigating bacterial proliferation in skin wounds and impacting the wound-healing cascade, it is definitely a therapeutic target of interest to address the problem of MDR infection.

There are several challenges to targeting NOD2 therapeutically, safely, and specifically. Stimulation of NOD2 responses by its ligand, muramyl dipeptide (MDP), has been demonstrated to induce broad-spectrum immune responses in mice that are protective against both bacterial and viral infections^15,36^. However, in humans, MDP has enhanced toxic, pyrogenic, and arthritogenic properties, precluding its use therapeutically^37^. This has led to the development of non-toxic MDP derivatives that retain select immunostimulatory activities, which are demonstrating benefit as an antiviral therapy in early stage clinical trials^38,39^. In our studies, there were no detected toxic effects of PALA on cellular viability, proliferation, or migration of primary human cells at concentrations that were found to be immunomodulatory (Fig. 2; Supplementary Movies 1 and 2). Additionally, we are targeting the NOD2 pathway with a small molecule compound, rather than a bacterial cell wall component that may be sensed by additional NLRs, such as NLRP3^15^. The compound PALA is a rationally-designed, transition-state inhibitor of the ATCase activity of CAD^25^, making it extremely specific to targeting this enzyme. The specificity of PALA to induce an antibacterial response through binding CAD was confirmed in this study (Fig. 7a) and in our previous study^16^ through RNAi-mediated knockdown of CAD. Interestingly, RNAi-mediated CAD knockdown blocked PALA-enhanced bactericidal activity, but it was not sufficient to induce the same level of AMP secretion from NHDF (Supplementary Fig. S1) or enhance the bactericidal activity of conditioned supernatants to the same degree as PALA treatment (Fig. 7b). These results may indicate that either the CAD knockdown was not efficient enough to induce as strong of a response as PALA treatment, or that the underlying molecular mechanisms involved are complex (i.e. differential requirements of CAD protein expression for NOD2 subcellular localization to activate the antibacterial response and CAD enzyme activity to repress NOD2 activity).

Newer therapeutic approaches to MDR infections are focused on aspects of host-pathogen interaction, rather than solely targeting bacterial viability. These approaches include targeting mechanisms involved in pathogen virulence, such as neutralization of bacterial toxins, prevention of biofilm formation or altering bacterial genes to “disarm” or disable the bacteria, rather than killing them^29^. For example, antibodies targeting *S. aureus* α-toxin, an important mediator of MRSA virulence, increased survival of mice challenged with a range of clinical *S. aureus* isolates in a pneumonia model^40^ and one antibody (AR301/Aridis) has already completed testing in Phase I/II human clinical trials^41^. Another promising approach targets quorum-sensing to impair the production of bacterial virulence factors, which has shown efficacy in a pre-clinical model of MRSA wound infection^42^. By disarming pathogens, these approaches make bacteria more sensitive to antibiotics and combination therapy results in synergistic effects, even on bacteria that are resistant to antibiotic monotherapy^42^. Interestingly, studies with MDP-derivatives have also demonstrated a synergistic effect with antibiotics, such as cefmenoxime, to clear infections with *Klebsiella pneumonia*^43^. As PALA does not have direct bactericidal effects (Fig. 1c), but rather mediates its effects through NOD2 signaling (Fig. 7e–g); it is anticipated that its antibacterial activity could also synergize with other antimicrobial agents that directly target bacteria (i.e. antibiotics, silver compounds, etc.), increasing their utility in MDR infections.

Exogenous application of small cationic peptides like cathelicidin and defensins, also known as “natural antibiotics” have also been shown to be efficacious in various infection models^14^. This is highlighted by studies examining the role of cathelicidin in control of bacterial infection and colonization. *Cnlp*-null (cathelicidin-deficient) mice demonstrated decreased clearance of group A *Streptococcus*^44^, while increased colonization of *S. epidermidis* was observed in a porcine wound model after inhibition of the active form of cathelicidin by neutrophil elastase cleavage^45^. Due to their important role in antimicrobial defense, pathogenic bacteria have evolved multiple mechanisms to decrease host production or activity of AMPs^46^. This has led to additional immune targeting therapeutic approaches to counteract pathogen-induced downregulation of AMPs and preserve host defenses^47,48^. This approach is now being studied in clinical trials, with a Phase II trial underway testing the efficacy of vitamin D supplementation on MRSA colonization^49^ and a successful Phase II trial reported for the treatment of shigellosis with sodium butyrate^47,50^. Recent studies have also focused on targeting *S. aureus* infections using various synthetic antimicrobial peptides^51,52^ and some of these peptides were shown to be effective against biofilms^52^. The results of our study also demonstrate the induction of an antibacterial response that targets multiple members of the ESKAPE pathogen family by PALA in both NHDFs and skin explants (Figs 3 and 4). Keratinocytes comprising the skin epidermis express HBD1-4. Of the four beta-defensins, HBD-1 is constitutively expressed in the skin and HBD2/HBD3 are released in response to bacterial infection^53^. On the other hand, cathelicidin (LL-37) is expressed by multiple cell types including neutrophils, mast cells, eccrine glands and keratinocytes as its inactive form hCAP 18^53,54^. In human skin, hCAP 18 is processed to form LL-37 by stratum corneum enzymes and proteases and is inducible by wounding and inflammation^53,55^. hCAP 18/LL-37 has been shown to be predominantly present in the granular and spinous layer of the epidermis^53,54^. AMPs exhibit broad spectrum antimicrobial activity and also possess the ability to modulate the host immune response. In this study we observed an increase in the production of both HBD2 and LL-37 protein upon infection of skin explants with MRSA and PALA treatment (Fig. 5) with no significant differences observed in HBD3 protein production. This result was consistent with the upregulation in genes encoding HBD2 and LL-37 observed in NHDF cells upon PALA treatment (Fig. 3c). While LL-37 levels were significantly increased in the human skin explant model in the differentiating keratinocyte layers of the skin, we observed no differences in LL-37 production in the conditioned media from fibroblasts (Fig. 3b). This may be due to technical difficulties in detecting secreted LL-37 *in vitro*, as another study was only able to detect LL-37 protein produced by cultured keratinocytes after immunoprecipitation of whole cell extracts; the levels in conditioned media were not detectable^54^. This was attributed to degradation of LL-37 upon secretion, presence of LL-37 binding proteins, or protease inhibitors in the media that interfered with production of mature LL-37^54^. Additionally, cell type response differences, cross-talk between various cell types in the skin epidermis^8^, or the complexity of the cytokine/chemokine milieu in response to infection all possibly contribute to the differences we observe between our *in vitro* fibroblast cultures and human skin explant model. The enhanced antibacterial defense in this study is potentially mediated, at least in part, through increased production of AMPs (Figs 3 and 5). Therefore, we predict that *in vivo* PALA treatment may act to maintain expression of AMPs during infection to promote bacterial clearance, rather than enhance inflammation.

To summarize, this study demonstrated that the pyrimidine synthesis inhibitor, PALA, derepresses the NOD2 signaling pathway and enhances antimicrobial, innate immune responses in human *in vitro* and *ex vivo* skin systems to increase clearance of ESKAPE pathogens. This approach could potentially be used alone or as adjunctive therapy to increase the efficacy of other antimicrobial treatments and help patients with wounds infected with bacterial strains non-responsive to traditional antibiotic treatments. This strategy to target antibiotic resistant bacteria through priming of the innate immune response is an important step towards designing new strategies to kill bacteria without increasing the burden of antibiotic resistance.

## Methods

### Reagents, Antibodies, and Plasmids

*N*-phosphonacetyl-l-aspartate (PALA, NSC224131) was obtained from the National Cancer Institute (NCI)/Division of Cancer Treatment and Diagnosis (DCTD)/Developmental Therapeutics Program (DTP) Open Chemical Repository (http:dtp.cancer.gov). Aquaphor® and Neosporin® were purchased from Target (Cleveland Heights, OH). Gentamycin solution was purchased from Sigma (St. Louis, MO). Rabbit monoclonal anti-CAD antibody (EP711Y) was purchased from Novus Biologicals (Littleton, CO). Rabbit polyclonal anti-RICK (H-300) was purchased from Santa Cruz Biotechnology (Santa Cruz, CA). Rabbit monoclonal anti-GAPDH (14C10) was purchased from Cell Signaling Technologies (Danvers, MA). Mouse monoclonal anti-α-tubulin (T5168) was purchased from Sigma. Antibodies against LL-37 (sc-166770) and HBD2 (sc-20798) were purchased from Santa Cruz Biotechnology. Anti-HBD3 antibody was purchased from Novus Biologicals (NB200-117). The plasmids, pcDNA3-HA-NOD1, pcDNA3-HA-NOD2, pBVIII-Luc, and pCMV-βgal were gifts of Gabriel Nuñez (University of Michigan) and have been previously described^56^. MISSION short hairpin RNA (shRNA) constructs targeting CAD (NM_004341.3-6910s21c1), NOD2 (NM_022162.1-2959s1c1), RIP2 (NM_003821.5-2364s21c1), and non-targeting shRNA control (SHC002) were purchased from Sigma.

### Bacterial strains

*S. aureus* Reynolds capsular serotype 5 (CP5)^57^ was a gift of Michelle Longworth (Cleveland Clinic). The clinically-derived, community-acquired, methicillin-resistant *S. aureus* (MRSA) strain MW2 (SAP227)^26^ with a stable chromosomal integration of the *lux* operon from *Photorhabdus luminescens*, was a gift of Roger Plaut (FDA). *P. aeruginosa* GFP (ATCC 10145GFP) and *A. baumanni* 5377 (ATCC 17978) strains were purchased from the American Type Culture Collection (ATCC; Manassas, VA). *S. aureus* and *A. baumanni* strains were grown in Tryptic Soy Broth (TSB) at 37 °C with aeration. *P. aeruginosa* was grown in Luria Bertani (LB) broth at 37 °C with aeration. Mid-log phase cultures were prepared by growing 1:7 dilutions of overnight cultures for 90 minutes at 37 °C with aeration.

### Cell Lines

The human embryonic kidney 293-HEK293T/17 (CRL-11268) cell line was purchased from ATCC and cultured in Dulbecco’s Modified Eagle Medium (DMEM) supplemented with 10% fetal bovine serum (FBS; Life Sciences, Carlsbad, CA). Normal adult human dermal fibroblasts (NHDF; CC-2511) were purchased from Lonza (Allendale, NJ) and cultured in FGM-2 media (CC-3132; Lonza). NHDF were transfected with shRNA contructs using a Nucleofector (Lonza) and the dermal fibroblast transfection kit (VPD-1001) according to manufacturer’s recommendations and assayed 48 h post-transfection. HEK293T/17 cells were transfected with expression constructs using Polyfect (Qiagen, Valencia, CA) according to manufacturer’s instructions and treated with 25 µM PALA 48 h post-transfection for collection of conditioned supernatants for bactericidal assays. Cell viability was determined by 2,3-Bis-(2-Methoxy-4-Nitro-5-Sulfophenyl)-2H-Tetrazolium-5-Carboxanilide (XTT) assay (Roche, Indianapolis, IN) and lactate dehydrogenase (LDH) secretion (Fisher Scientific, Waltham, MA) according to manufacturer’s instructions.

### *In Vitro* Scratch Wound Assay

NHDF were plated in removable culture 2 well inserts (ibidi; Munich, Germany) at 24,000 cells/insert well in triplicate. After 24 hours, the inserts were removed and fresh media added with or without 25 µM PALA. Automated time-lapse microscopy was performed using a Leica DMI6000 inverted microscope with LAS X software (Leica Microsystems, GmbH; Wetzlar, Germany) and images captured every 15 minutes for 27 hours by a Hamamatsu Orca Flash4 camera (Hamamatsu Photonics; Shizuoka, Japan). Image analysis was performed using a custom macro in ImagePro Plus software (Media Cybernetics; Rockville, MD).

### RNA Analysis

NHDF cells treated with or without 25 μM PALA in triplicate for 16 hours were collected and RNA isolated using the Qiagen RNeasy Mini Kit. RNA was converted to cDNA using the BioRad iScript cDNA synthesis kit. qPCR was performed in duplicate using SYBR Green supermix and quantified using the 2^−ΔΔCT^ method^58^. Primers targeting *DEFB4A* (HBD2), *DEF103B* (HBD3), *CAMP* (cathelicidin), and *NOD2* are listed in Supplementary Table S1.

### Luciferase Reporter Assays

HEK293T/17 cells were plated at 10,000 cells/well in 48 well plates in triplicate for transfection using Polyfect (Qiagen) according to manufacturer’s protocol. Transfected cells were infected 24 hours post-transfection with *S. aureus* CP5 at a multiplicity of infection (MOI) of 5 for 6 hours. Cells were collected in 1× reporter lysis buffer (100 µL/well; Promega, Madison, WI) and subjected to two freeze/thaw cycles. Luciferase and β-galactosidase activity measured as previously described^59^ and presented as luciferase activity normalized to transfection efficiency determined by β-galactosidase levels.

### Immunoblot Assays

Cells were washed 2× in PBS, then lysed in Ginger buffer (310 mM Tris pH 6.8, 25% glycerol, 5% SDS, 715 mM 2-mercaptoethanol, 125 mg/mL bromphenol blue) on ice for 10 minutes. The resulting lysate was separated by SDS-PAGE and proteins transferred to a polyvinylidene fluoride membrane using the iBlot2 system (ThermoFisher Scientific; Cleveland, OH). Membranes were blocked in 5% milk/TBST (Tris buffered saline, 0.1% Tween-20) and then probed with primary antibodies overnight at 4 °C. After washing with TBST, blots were probed with horseradish peroxidase-conjugated secondary antibodies for 1 h in 2.5% milk/TBST. Blots were developed by enhanced chemiluminescence. Uncropped scans of immunoblots used to create Fig. 7 are included in Supplementary Fig. S2.

### NHDF Infection

NHDF were seeded at 20,000 cells/well in duplicate in 24 well plates in FGM-2 media the day before treatment. Media was changed to DMEM + 10% FBS and stimulated with PALA (1–25 µM) for 1 h prior to infection with SAP227 (MOI = 5) for 2 hours. Bioluminescence of SAP227 was monitored at 30 minute intervals for 10 s/well in a Flexstation 3 (Molecular Probes, Eugene; OR). After 2 h, supernatants were collected and cells lysed in 50 µL 1%Triton X-100/PBS on ice for 10 minutes. Cell lysates and supernatants were combined and viable bacteria determined through serial dilution and plating of duplicate samples on LB agar plates.

### NHDF Conditioned Supernatant Bactericidal Activity Assays

NHDF were plated at 20,000 cells/well in 24 well plates in FGM-2 media. The next day, the media was changed to DMEM + 10% FBS and cells were stimulated with PALA (1–25 µM) for 16 hours. Cell supernatants were collected and cell debris was removed by centrifugation (500x*g*, 5 minutes). Antibacterial activity was assessed in triplicate by combining 50 µL of conditioned media with 100,000 cfu for 1–2 h in a total volume of 100 µL. Bacterial viability was determined through serial dilution and the plating of duplicate samples on LB agar plates. AMP levels in conditioned media was determined by ELISA for HBD2 (Peprotech, Rocky Hill, NJ), HBD3 (Peprotech), and cathelicidin (LL-37; Hycult, Plymouth Meeting, PA) according to manufacturer’s protocols. NHDF transfected with shRNA constructs were plated at 33,000 cells/well in 24 well plates in FGM-2 media. The next day, the media was changed to DMEM + 10% FBS and the cells were stimulated with PALA (25 µM) for 24 hours. Cell supernatants were collected and antibacterial activity was assessed in triplicate by combining 170 µL of conditioned media with 100,000 cfu for 1 h in a total volume of 180 µL. Bacterial viability was determined through serial dilution and the plating of duplicate samples on LB agar plates.

### Skin Explants

Skin was excised from the back or upper buttocks of Yorkshire pigs according to relevant regulatory guidelines and regulations in a protocol approved by the Cleveland Clinic Institutional Animal Care and Use Committee (IACUC#2014-1341). De-identified residual/redundant human skin tissue was collected from individuals undergoing elective breast reduction, breast reconstruction surgery, thighplasty, facelift surgery or abdominoplasty at the Cleveland Clinic under a protocol approved by the Cleveland Clinic Institutional Review Board (IRB#13-1381) in accordance with the relevant regulatory guidelines and regulations. Underlying fat and fascia were removed by scalpel and then the skin was rinsed twice in PBS. Skin samples were washed in DMEM + 2 × penicillin/streptomycin/Amphotericin B for 1 h at 37 °C. The samples were placed in DMEM + 10% FBS and allowed to rest overnight at 37 °C. The following day, biopsies (6 mm) were cut, the epidermal layer perforated three times either with a sterile 25 g needle (pig) or GREER allergy test needles (human), and placed on Transwell inserts (6 µM pore size) in 24 well plates with DMEM + 10% FBS in the lower chamber or gauze sponge in 6 well plates with DMEM + 10% FBS. The apical surface was infected with SAP227, *P. aeruginosa*, or *A. baumannii* (3 million cfu/explant). After 1 h, Aquaphor, 2%(w/w) PALA in Aquaphor (Cleveland Clinic Investigational Drug Service), or Neosporin was applied to the apical surface in triplicate and incubated for an additional 4 hours or 24 hours. Tissue was homogenized in 2 mL PBS using an Omni-GLH homogenizer (Thomas Scientific, Swedesboro, NJ) and bacterial viability determined in serial dilutions plated in duplicate on LB agar plates. Samples infected with SAP227 and treated with Aquaphor, 2% PALA, or Neosporin were embedded for immunohistochemistry and homogenized for ELISA assays. AMP levels in tissue homogenates was determined by ELISA for HBD2 (Peprotech, Rocky Hill, NJ), HBD3 (Peprotech), and cathelicidin (LL-37; Hycult, Plymouth Meeting, PA) according to manufacturer’s protocols.

### Immunohistochemistry

Infected human skin explants treated with Aquaphor or 2% PALA were fixed in Histochoice. Next, paraffin embedded samples were sectioned and stained for HBD2, HBD3, or LL-37. Briefly, dewaxed and rehydrated sections were first subjected to antigen retrieval in sodium citrate buffer using a steamer for 25 minutes. Primary antibody staining was performed at 4 °C overnight in a humidifying chamber. Labelled polymer-HRP anti-rabbit (Dako, K4011), biotinylated secondary goat anti-mouse (EMD Millipore, IHC Select, 20775), and streptavidin-HRP (EMD Millipore, IHC Select, 20774) were used as detection reagents/secondary antibodies. Vina green chromogen (Biocare Medical, BRR807A) was used to visualize protein levels. Aperio AT2 (Leica Biosystems) was used to scan the slides.

### Statistical analyses

All statistical analyses were performed on GraphPad Prism software (Version 5.02). One-way or two-way Analysis of Variance (ANOVA) with post-hoc Bonferroni or Tukey’s multiple comparison test was used to determine significance of datasets with multiple variables. For datasets with two variables, unpaired, two-tailed, Students t-test or Wilcoxon matched-pairs signed rank test was used to determine significance. Details of tests used for specific experiments have been included in the figure legends. Results are representative of a minimum of three independent experiments performed in triplicate.

### Data availability

All data generated or analyzed during this study are included in this published article and its supplementary information files.

## Electronic supplementary material

Supplementary Information

Supplementary Movie S1

Supplementary Movie S2
