# Supplementary material for: Pyrimidine synthesis inhibition enhances cutaneous defenses against antibiotic resistant bacteria through activation of NOD2 signaling
Source: Sci Rep. 2018 Jun 7;8:8708. doi: 10.1038/s41598-018-27012-0 (PMC5992176; doi:10.1038/s41598-018-27012-0)

Supplementary Information

Pyrimidine synthesis inhibition enhances cutaneous defenses against antibiotic resistant bacteria through activation of NOD2 signaling.

Samreen Jatana, Craig R. Homer, Maria Madajka, András K. Ponti, Amrita Kabi, Francis Papay & Christine McDonald

Supplementary Table S1: Primers used for qRT-PCR

Protein	Gene	Forward Primer	Reverse Primer
HBD2	*DEFB4A*	5'-GTGAAGCTCCCAGCCATCAGCCAT	5'-AGCAGCTTCTTGGCCTCCTCATG
HBD3	*DEFB103B*	5'-GCGTGGGGTGAAGCCTAGCA	5'-AGCTGAGCACAGCACACCGG
Cathelicidin	*CAMP*	5'-GGAAGCTGTGCTTCGTGCTA	5'-TCTGGGTCCCCATCCATCGT
NOD2	*NOD2*	5'- AAATCAGGTTGCCGATCTTCA	5'- CAGCCAATCCATTCGCTTTC

Supplementary Figure S1: AMP levels in conditioned media from NHDF with and without CAD expression knocked-down by RNAi. NHDF were transfected with non-targeting (shControl) or CAD-targeting (shCAD) shRNA constructs. After 48h, cells were treated with 25µM PALA (16 hours) and conditioned media collected. Protein levels of secreted HBD2 and HBD3 in conditioned media was determined by ELISA. Mean±SD, n=3-5 independent experiments; significance determined by 2-way ANOVA with Bonferroni multiple comparisons test; *$p<0.05$.

Supplementary Figure S2: Scans of uncropped immunoblot films with areas used for Figure 5 boxed. (a) CAD immunoblot of NHDF transfected with non-CAD targeting shRNA or CAD targeting shRNA used in Figure 5a. **(b)** Tubulin loading control immunoblot for Figure 5a. **(c)** RIP2 immunoblot of NHDF transfected with a non-targeting shRNA (shC) or RIP2-targeting shRNA (shRIP2) used in Figure 5c. **(d)** GAPDH loading control immunoblot for Figure 5c.

Supplementary Movie S1: Migration of untreated NHDF. Time lapse movie of migration of untreated NHDF into a 500µm gap over 27 hours. Frames taken every 15 minutes for a total of 27 hours. Representative of 3 independent experiments performed in triplicate.

Supplementary Movie S2: Migration of PALA-treated NHDF. Time lapse movie of migration of NHDF in the presence of 25µM PALA into a 500µm gap over 27 hours. Frames taken every 15 minutes for a total of 27 hours. Representative of 3 independent experiments performed in triplicate.

Supplementary Figure S1

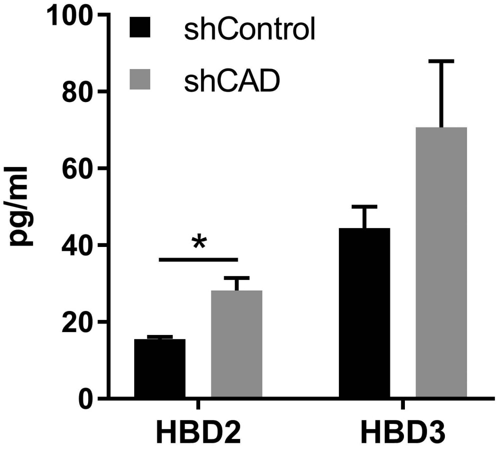

Supplementary Figure S2

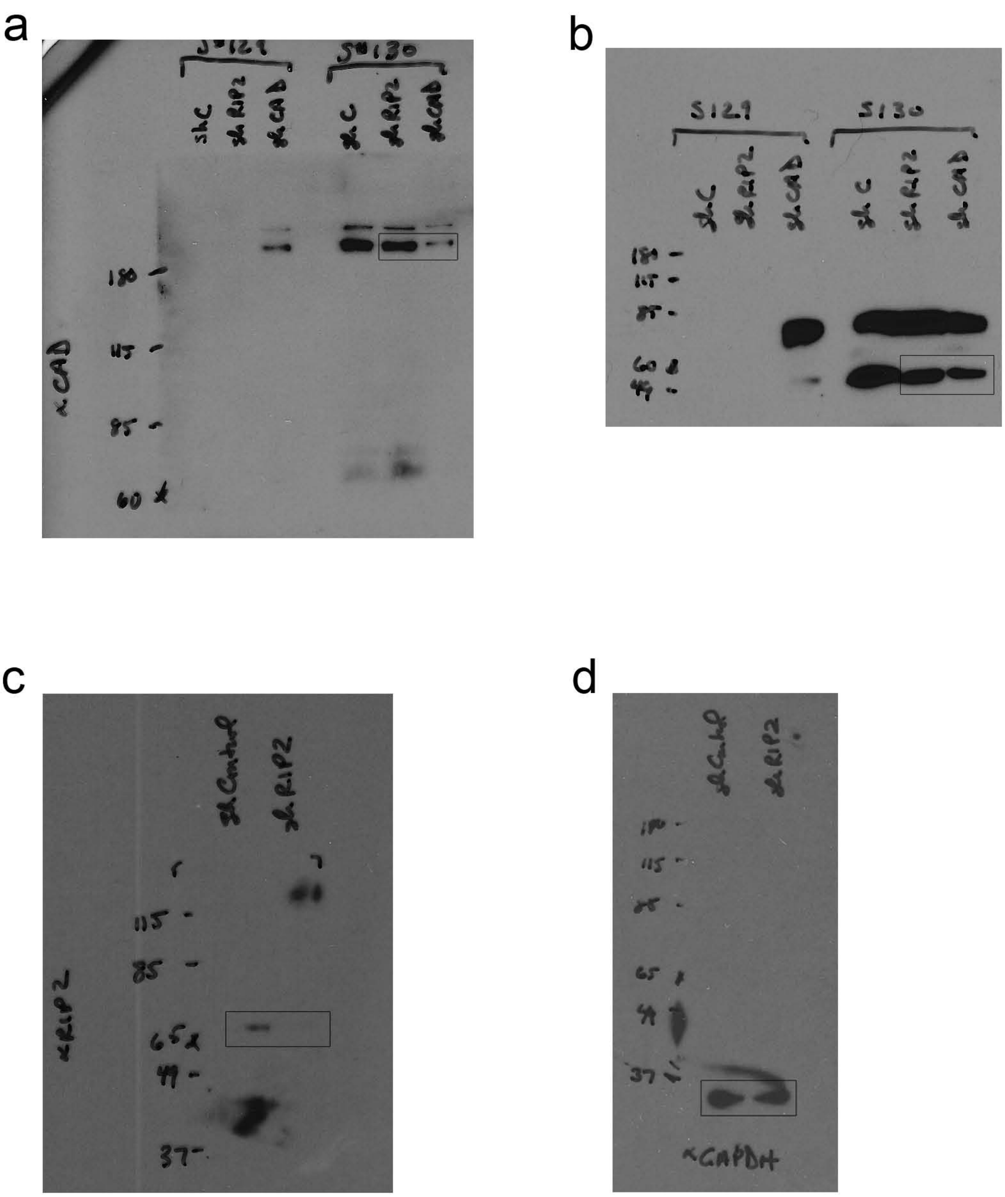

Supplement: Supplementary file 1 — Supplementary Information [file 41598_2018_27012_MOESM1_ESM.pdf]
